# Association between *ELP4* rs986527 polymorphism and the occurrence and development of intracranial arachnoid cyst

**DOI:** 10.1002/brb3.1480

**Published:** 2019-11-19

**Authors:** Kai Li, De‐Sheng Kong, Jun Zhang, Xin‐Sheng Wang, Xun Ye, Yuan‐Li Zhao

**Affiliations:** ^1^ Department of Neurosurgery Beijing Tian Tan Hospital Capital Medical University Beijing China; ^2^ Department of Neurosurgery Peking University International Hospital Beijing China

**Keywords:** *ELP4*, incidence, intracranial arachnoid cyst, polymorphism

## Abstract

**Objective:**

The association between ELP4 rs986527 polymorphism and the occurrence and development of intracranial arachnoid cyst was studied in this paper.

**Methods:**

Eighty‐five patients diagnosed with intracranial arachnoid cysts by cerebral computed tomography scan were selected. Sixty‐three healthy volunteers for medical examination in hospitals served as controls. The cognition, depressive symptoms, and the likelihood of headache, dizziness, head trauma history, dementia, depression, and epilepsy were assessed. ELP4 genotypes and its allele frequency were determined by PCR, endonuclease restriction analysis, and gel electrophoresis.

**Results:**

ELP4 rs986527 had three genotypes: TT, TC, and CC. The intracranial arachnoid cyst group showed no statistically significant difference in genotype frequencies compared with healthy controls. There was no significant correlation between ELP4 rs986527 polymorphism and location of intracranial arachnoid cyst. TC and C genotype frequencies were associated with a higher incidence of clinical symptoms than TT genotype frequencies, and C allele frequencies were associated with a significantly higher incidence of clinical symptoms compared with T allele frequencies. There was no significant difference in TNF‐α and IL‐1β levels between TT/TC/CC genotypes before treatment. After treatment, the levels of TNF‐α and IL‐1β were significantly decreased in different genotypes, and the decrease in CC was the greatest. The frequency of TT and TC genotypes was higher than that of CC genotypes.

**Conclusion:**

ELP4 rs986527 polymorphism affected the incidence of clinical symptoms and the levels of TNF‐α and IL‐1β in patients with intracranial arachnoid cysts.

## Graphical abstract

Intracranial arachnoid cysts with a high prevalence. In this paper, we investigated the association between elongator protein complex 4 (ELP4) rs986527 polymorphism and the occurrence and development of intracranial arachnoid cysts.

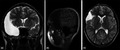

## 1 INTRODUCTION

The arachnoid membrane is the last meninges that differentiate during fetal development (Liao et al., 2018). Arachnoid cysts may occur when cerebrospinal fluid flow changes in the early stages of subarachnoid space formation leading to rupture of the developing arachnoid membrane (Zhang et al., 2018). The best diagnostic clue for arachnoid cysts is a clearly defined external axis cyst that can displace or deform adjacent brains (Hong et al., 2017). Intracranial arachnoid cysts are congenital malformations characterized by abnormal cerebrospinal fluid accumulation in the cerebral cistern and major brain fissures in the arachnoid and subarachnoid spaces (Chen et al., 2017). Intracranial arachnoid cysts are relatively common entities, accounting for approximately 1% of all intracranial space‐occupying lesions (Hsu & Lee, 2017). The study showed that the prevalence of intracranial arachnoid cysts in people over the age of 45 was 1.1%, and the prevalence was 1.7% in healthy young men (Park, Shin, & Lee, 2017). The prevalence rate was found to be 0.5%–1.7% in hospitals and clinical studies, and the male incidence rate was two to three times higher than that of females (Kwiatkowska, Hałabuda, & Rybus, 2019). The prevalence of intracranial arachnoid cysts in children has recently been reported to be 2.6%. Intracranial arachnoid cysts consist of arachnoid fluid‐filled cysts that usually do not communicate with the ventricular system, most of which are accidentally discovered and generally asymptomatic (Alg et al., 2018). However, some patients are symptomatic and need neurosurgery (Gan, Liu, & Hu, 2017). Symptoms of intracranial arachnoid cysts may include headache/increased intracranial pressure, hydrocephalus, localized mass effects, or cyst rupture (Meng, Hao, & Zhao, 2019). Local mass effects may contribute to local neurological deficits depending on adjacent neural structures or bone anatomy. These lesions may also rupture, leading to subdural hematomas or hemorrhage (Sima, Sun, & Zhou, 2017). In this paper, we investigated the association between elongator protein complex 4 (*ELP4*) rs986527 polymorphism and the occurrence and development of intracranial arachnoid cysts.

## 2 MATERIALS AND METHODS

### 2.1 Patients

Patients with intracranial arachnoid cysts who were treated at our hospital from May 2017 to September 2018 were selected as subjects of this study. Eighty‐five patients with intracranial arachnoid cysts had a mean age of 5.4 ± 1.3 years, including 49 (57.65%) male patients and 36 (42.35%) female patients. In addition, 63 healthy control participants were selected, with an average age of 5.3 ± 1.3 years, including 36 (57.14%) male patients and 27 (42.86%) female patients. There was no significant difference in gender, age at diagnosis, and body mass index between the intracranial arachnoid cyst group and the healthy control group.

Patients, regardless of gender, diagnosed with intracranial arachnoid cysts were included. All patients provided written informed consent. Patients with focal atrophy, postoperative or postischemic lesions, or other lesions that may mimic cystic arachnoid cysts were excluded.

The study protocol was approved by the Medical Research Ethics Committee. Informed consent was obtained from participants or their guardians, blood samples were collected, and DNA was prepared using standard techniques.

### 2.2 Methods

#### 2.2.1 Radiological assessment

Intracranial arachnoid cysts were examined by magnetic resonance imaging (MRI). Three observers conducted a preliminary assessment of all scans, including a neurosurgery resident, a medical student, and a clinical neurology professor. All patients with suspected arachnoid cysts or those found to be unrecognized were reassessed by a senior neuroradiologist who underwent a final diagnosis of arachnoid cyst according to the following criteria: The quality of the homogeneous subarachnoid cystic lesion on the surrounding tissue was affected by compression. Radiologists were blinded to clinical data. Patients with focal atrophy, postoperative or postischemic lesions, or other lesions that may mimic cystic arachnoid cysts were excluded.

#### 2.2.2 Clinical evaluation

Clinical assessment was conducted by a psychiatrist and a specially trained psychiatric research nurse and included semistructured neuropsychiatric examinations and interviews with key informants. Headache was defined as having any type of headache in the past 3 months. Dizziness was defined as having dizziness in the past 3 months and had imbalance or instability or the usual symptoms of any of these symptoms. Head trauma was defined as a noteworthy head trauma with or without confusion, and with or without hospitalization. Cognition was measured using the Mini‐Mental State Examination, and depression was assessed using the Montgomery–Åsberg Depression Rating Scale. As mentioned earlier, psychiatrists diagnose dementia based on DSM‐III‐R and DSM‐5. The frequency of epilepsy was based on interviews and data from the patient registry.

#### 2.2.3 ELP4 rs986527 polymorphism genotyping

DNA was extracted from blood using the Puregene DNA Isolation Kit (Gentra). *ELP4* genotype was determined by PCR, endonuclease restriction analysis, and gel electrophoresis. The PCR was carried out in a volume of 20 μl containing 50 ng of DNA, 16 pmol of each primer, 200 μM of each dNTP, 50 mM KCl, 10 mM Tris‐HCl (pH 8.3), 2.0 mM MgCl_2_, and 1.0 U AmpliTaq Gold (Applied Biosystems). *Taq* DNA polymerase (Thermo scientific Cat no: #EPO402) was dispensed into each reaction tube, and then, 2 μl of DNA sample was added to each sample. The PCR was run as follows: initial denaturation at 95°C for 5 min, denaturation at 95°C for 30 s, annealing at 67°C for 30 s, and extension at 72°C for 1 min for a total of 36 cycles with a final extension at 72°C for 5 min. A 3% agarose gel was prepared for running the samples. Agarose gel electrophoresis was first performed to control amplification of the PCR product, and then, the allele was detected after RFLP. Endonuclease restriction analysis was performed using 1 μl of PCR product and 1 μl of enzyme digestion buffer (10 mM Tris‐HCl, 10 mM MgCl 2, 50 mM NaCl, 1 mM dithiothreitol [pH 7.9, 25°C], 2 U *Bse*RI and 7.5 μl of deionized water). The reaction mixture was incubated for 2 hr at 37°C and then incubated for 20 min at 65°C. The digested samples were resolved on a 3% agarose gel and scanned with a UV transilluminator. Targeting SNP was studied by identifying restriction enzymes in this region, and the alleles were identified. RT‐PCR primer sequences are shown in Table 1.

**Table 1:** RT‐PCR primer sequences

Gene	Upstream primer	Downstream primer
*rs986527*	CGCTAGGTACGCTACGTTAA	GGTTCGCGCTAATTAATATGC

### 2.3 Statistical analysis

Continuous variables were compared using an independent sample *t* test and the Mann–Whitney *U* test. Pearson's chi‐square test or Fisher's exact test was used to compare the data in appropriate circumstances. The classification data were compared using the Cochran–Mantel–Haenszel test. Mortality was compared by using Kaplan–Meier survival curves and log‐rank tests. Statistical significance was determined by *p* value <.05. All analyses were performed using SPSS version 20.0 (IBM).

## 3 RESULTS

### 3.1 Demographic and baseline characteristics of the study population

The study included 85 patients with intracranial arachnoid cysts (children with intracranial arachnoid cysts, 49 males and 36 females) and 63 healthy control participants (healthy children, 36 males and 27 females). There were no significant differences in gender, age at diagnosis, and body mass index between the groups (*p* > .05). Influence of these factors on the test results was excluded, and the data were comparable. Compared with the healthy control group, the blood routine indexes of the intracranial arachnoid cyst group were not significantly different (*p* > .05; Table 2).

**Table 2:** Demographic and baseline characteristics of the study population

Variables	Healthy control	Intracranial arachnoid cyst	Chi‐square value	*p* Value
Diagnostic age	5.27 ± 1.33	5.42 ± 1.25	2.154	.587
Gender: male or female	36:27	49:36	3.264	.695
BMI	22.14 ± 2.44	22.85 ± 1.58	1.556	.387
White blood cells (×10^9^/L)	8.17 ± 0.54	8.63 ± 0.44	6.854	.652
Red blood cells (×10^12^/L)	4.62 ± 0.55	4.53 ± 0.38	5.335	.473
Hemoglobin (g/L)	138.28 ± 15.95	135.47 ± 22.17	4.156	.587
Lymphocytes (l%)	0.35 ± 0.08	0.34 ± 0.06	3.573	.289
Platelet (×10^9^/L)	264.15 ± 22.54	258.17 ± 18.67	4.257	.542

### 3.2 Clinical examination of patients with intracranial arachnoid cyst

Clinical examination of patients with intracranial arachnoid cysts was performed, including 22 patients with headache/dizziness (25.88%), 11 patients with head trauma (12.94%), 25 patients with epilepsy (29.41%), eight patients with hip fracture (9.41%), 16 people with dementia (18.82%), and 32 people with depression (37.65%; Table 3).

**Table 3:** Clinical examination of patients with intracranial arachnoid cyst

Variables	*n*	Proportion
Headache/dizziness	22	25.88%
Head trauma history	11	12.94%
Epilepsy	25	29.41%
Hip fracture	8	9.41%
Dementia	16	18.82%
Depression	32	37.65%

### 3.3 MRI examination of fenestration for the treatment of intracranial arachnoid cysts

**Figure 1:**
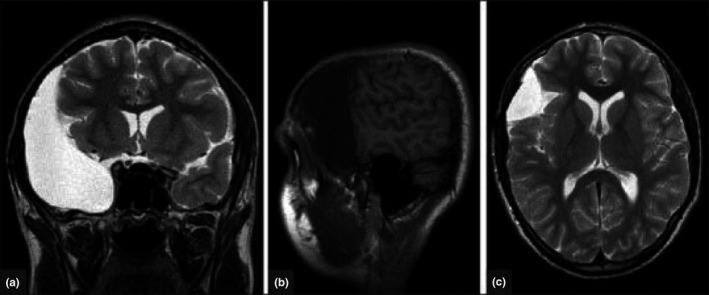
MRI examination of bladder fenestration for treatment of intracranial arachnoid cysts

In the right side, anterior cranial fossa arachnoid cyst was surgically treated for traumatic edema. Despite attempts to manage the development of edema through the bladder peritoneal shunt, the patients were symptomatic, requiring a fenestration of the wall and regular follow‐up. Coronal T2‐weighted MRI performed at 3 years of follow‐up showed reabsorption of right hemisphere edema (a). Sagittal T1‐weighted MRI showed a reduction in the mass effect caused by arachnoid cysts associated with re‐expansion of the sulci (b). The axis T2‐weighted MRI showed resolution of the midline shift and the relief of lateral ventricle compression (c; Figure 1).

### 3.4 ELP4 rs986527 genotype and allele frequency distribution

**Figure 2:**
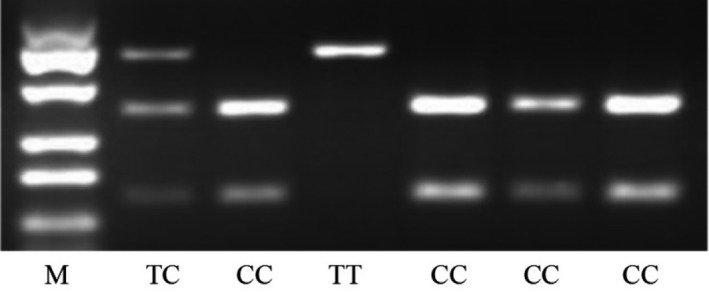
*ELP4* rs986527 polymorphism

Genotyping of *ELP4* rs986527 polymorphism was performed in all intracranial arachnoid cyst patients and healthy controls using genomic DNA extracted from peripheral blood leukocytes. The genotype of all patients was analyzed by GeneChip Mapping NspI 250 K SNP Arrays (Affymetrix), and the parameter linkage analysis was performed by Merlin software. There were three genotypes in *ELP4* rs986527: TT, TC, and CC. There were no significant differences in TT, TC, and CC genotype frequencies and T and C allele frequencies in intracranial arachnoid cysts, compared with the healthy controls (*p* > .05; Figure 2, Table 4).

**Table 4:** *ELP4* rs986527 genotype and allele frequency

Project	*n*	TT (%)	TC (%)	CC (%)	T (%)	C (%)
Healthy control	63	15 (23.81)	30 (47.62)	18 (28.57)	47.62	52.38
Intracranial arachnoid cyst	85	20 (23.53)	40 (47.06)	25 (29.41)	47.06	52.94
Chi‐square value	—	4.752	3.654	2.584	1.257	6.389
*p* Value	—	.425	.336	.528	.189	.257

### 3.5 *ELP4* rs986527 polymorphism and intracranial arachnoid cyst site

The relationship between *ELP4* rs986527 polymorphism and intracranial arachnoid cysts was studied. The results showed that TT, TC, and CC genotypes were not significantly correlated with location of intracranial arachnoid cysts (*p* > .05; Table 5).

**Table 5:** *ELP4* rs986527 polymorphism and intracranial arachnoid cyst site

*ELP4* rs986527	*n*	Middle cranial fossa	Brain convex	Quadruple pool
TT	20	12 (60.00%)	6 (30.00%)	2 (10.00%)
TC	40	24 (60.00%)	13 (32.50%)	3 (7.50%)
CC	25	15 (60.00%)	8 (32.00%)	2 (8.00%)
*F* value	—	15.478	12.367	11.125
*p* Value	—	.674	.235	.158

### 3.6 *ELP4* rs986527 polymorphism and clinical symptom rate

The incidence of polymorphism and clinical symptoms of ELP4 rs986527 in all patients with intracranial arachnoid cysts was studied. The results showed that the frequency of TC and CC genotypes was higher than that of TT genotype frequencies, and the C allele frequency was higher than the T allele frequency, and the difference was statistically significant (*p* < .05; Table 6).

**Table 6:** *ELP4* rs986527 polymorphism and clinical symptoms

ELP4 rs986527	*n*	Incidence rate	OR	95% CI
TT	14	3 (21.43%)	1.00	1.00
TC	37	12 (32.43%)	2.63	1.24–3.57
CC	34	15 (44.12%)	5.48	4.13–6.42
T	51	15 (29.41%)	1.00	1.00
C	71	27 (38.03%)	3.54	2.18–4.26

### 3.7 Effects of *ELP4* rs986527 polymorphism on inflammatory factors

The levels of TNF‐α and IL‐1β were not significantly different in TT/TC/CC genotypes before treatment. After treatment, the levels of TNF‐α and IL‐1β were significantly decreased in different genotypes (*p* < .05), and the decrease in CC was the greatest. The difference was statistically significant (*p* < .05; Table 7).

**Table 7:** Effect of *ELP4* rs986527 polymorphism on inflammatory factors

ELP4 rs986527	TNF‐α		IL‐1β	
	Before treatment	After treatment	Before treatment	After treatment
TT	2.64 ± 0.55	1.24 ± 0.25	3.54 ± 0.73	1.53 ± 0.18
TC	2.53 ± 0.47	1.37 ± 0.41	3.62 ± 0.84	1.92 ± 0.28
CC	2.72 ± 0.56	1.86 ± 0.34	3.57 ± 0.64	2.34 ± 0.36
*F* value	14.152	12.334	11.147	12.854
*p* Value	.418	.014	.598	.008

## 4 DISCUSSION

Arachnoid cysts are congenital and benign, and mostly asymptomatic, and require no special attention. The most common sites of arachnoid cysts include the middle cranial fossa, the posterior cerebellum, and the convex surface (Wang et al., 2018). Some intracranial sites of arachnoid cysts are gender‐dependent, with a higher incidence of sacral cysts in males and a higher incidence of cerebral horn cysts in women (Irfan, Rathore, & Karim, 2017). Treatments for arachnoid cysts include cyst‐peritoneal shunt, craniotomy, or endoscopic fenestration and stereotactic aspiration (Baldawa, Baldawa, & Baldawa, 2017). Igarashi Y *et al*. believed that advances in neurosurgical techniques and neuroendoscopy were conducive to fenestration cyst‐peritoneal shunt as a surgical option for treatment of arachnoid cysts (Igarashi et al., 2017). An intracranial arachnoid cyst is a benign developmental cyst containing cerebrospinal fluid, which is usually found by chance on MRI. These cysts are slowly growing and are usually asymptomatic. When symptoms appear, current features include focal neurological deficits, elevated intracranial pressure, or seizures (Berle & Wester, 2018). In the retrospective study of 867 epilepsy patients by Zakaria J, 17 (1.96%) patients had subarachnoid cyst (Zakaria, Wemhoff, & Anderson, 2018). In the study by Lechanoine, Spennato, and Ruggiero (2019), eight in 20 patients with epilepsy and associated arachnoid cysts were classified as idiopathic systemic or localized‐related epilepsy based on clinical and EEG characteristics.

*ELP4* is part of the multi‐subunit (ELP1‐ELP6) extension complex that regulates neuronal maturation. The extension plays a key role in transcription, regulating the actin cytoskeleton, cell movement, and migration genes (Jr, Mercier, & Sindou, 2018). These functions are critical in the nervous system for neuronal growth, cone movement, axon growth and guidance, neuronal migration during neurogenesis, and development (Walker, Gholamrezanezhad, & Bucklan, 2017). Interestingly, another type of extension subunit mutation is associated with human neurological disease. Riley‐Day syndrome (MIM 223900) is an autosomal recessive, sensory, and autonomic neuropathy with EEG abnormalities and epilepsy (Mishra, Pruthi, & Bharath, 2017). Riley‐Day syndrome is caused by mutations in the splice site of the *hELP1* (or *IKAP*) gene, which results in the expression of tissue‐specific exon skipping and truncated mRNA transcripts (Nikolić et al., 2017). Patients with Riley‐Day have reduced cell motility and can be rescued by wild‐type hELP1. *ELP4* mutations may also partially abolish the extension function in the central nervous system through its effects on a variety of cellular and actin cytoskeletal genes or proteins (French et al., 2017). The role of *ELP4* is shown by a genome‐wide linkage scan showing the electroencephalographic features of the central‐temporal spike. It has been reported that 38 families have developed a locus with a central‐sacral spike‐like trait at chromosome 11p13 with a maximum log score of 4.30. Subsequent fine mapping revealed the association of three intron single nucleotide polymorphisms (SNPs) within the *ELP4* gene. After linkage analysis of the family, it was found that the central‐sputum spike‐wave discharge of EEG was associated with two SNPs of *ELP4* rs964112 and rs986527. The alleles of rs964112 complementary strand were T and G, and the G allele improved the risk of spike‐wave discharge in the central‐temporal region of the EEG (OR = 1. 88, *p* < .001). The alleles of the complementary strand of rs986527 were T and C, and the C allele increased the risk of spike discharges in the central‐temporal region of EEG (OR = 1. 88, *p* < .001), and the two SNP loci were found to be extremely similar. In this study, compared with the healthy control group (23.81%, 47.62%, 28.57%, and 47.62%), there were no significant differences in genotype frequencies of TT (23.53%), TC (47.06%), and CC (29.41%) and allele frequencies of T (47.06%) and C (52.94%) in intracranial arachnoid cyst group. The frequency of TC (32.43%) and CC (44.12%) genotype was higher than that of the TT (21.43%) genotype, and the frequency of C (38.03%) allele was clinically higher than that of T (29.41%) allele. It indicated that *ELP4* was associated with the pathogenesis of intracranial arachnoid cysts, and *ELP4* rs986527 polymorphism can affect the incidence of clinical symptoms in patients with intracranial arachnoid cysts.

Arachnoid cysts are subarachnoid inflammatory reactions caused by brain trauma, cerebral hemorrhage, or intracranial infections, leading to pathological accumulation of cerebrospinal fluid, characterized by inflammatory cells and hemosiderin deposition. Pro‐inflammatory factors IL‐1β and TNF‐α are mainly secreted by macrophages and play an important role in the inflammatory response. They are the initiators of the inflammatory factor cascade and promote downstream inflammatory factors and cells by activating endothelial cell inflammatory signaling pathways. The release of adhesion molecules forms a positive feedback loop of the inflammatory response, exacerbating the occurrence of inflammatory reactions. This study found that TNF‐α and IL‐1β levels were not significantly different between TT/TC/CC genotypes before treatment, and TNF‐α and IL‐1β levels were significantly lower in patients with different genotypes after treatment (*p* < .05), and the decrease in CC was the greatest. The frequency of TT and TC genotypes was higher than that of CC genotypes. In addition, the author analyzed the clinical significance of ELP4. This study found that TC (32.43%) and CC (44.12%) genotype frequencies were higher than TT (21.43%) genotype frequencies, and C (38.03%) allele frequencies were higher than T (29.41%) allele frequencies. There was no significant difference in TNF‐α and IL‐1β levels between TT/TC/CC genotypes before treatment. The levels of TNF‐α and IL‐1β were significantly decreased in patients with genotype, and level in CC was the lowest.

In conclusion, our results indicated that *ELP4* was associated with pathogenesis of intracranial arachnoid cysts. *ELP4* rs986527 polymorphism affected the incidence of clinical symptoms and the levels of TNF‐α and IL‐1β in patients with intracranial arachnoid cysts. Arachnoid cysts had different localizations in the intracranial region. Further studies may reveal pathogenic genes or variants of the intracranial arachnoid cyst phenotype, clarify the pathogenesis of the disease, and, importantly, provide insights into meningeal development.

## CONFLICT OF INTEREST

None declared.

## Data Availability

The data that support the findings of this study are available from the corresponding author upon reasonable request.
